# Anæsthesia in Labor

**Published:** 1881-02

**Authors:** Dudley M. Culver

**Affiliations:** Whitesville, Indiana


### Selections.

---

### "ANÆSTHESIA IN LABOR."

By DUDLEY M. CULVER, M.D., WHITESVILLE, INDIANA.

This is a subject that is attracting the attention of the profession to no small extent at the present day, and it is a subject that demands statistics of actual cases to establish its efficacy or the opposite. For young men of to-day, just starting in the practice of medicine, have many traps easily ensnaring them, and who can they look to for counsel and authority but the old members of the profession?

Anæsthetics have been used just enough now in labor to make a vast number of women appeal to the tender compassion of the accoucheur to administer chloroform. And they will tell the young physician that Dr. —— did use it on Mrs. ——, and no bad results followed, and it will be hard for a young man to refuse when he is competing against a fearless man who does use it.

I read in a recent number of the *Gazette* with marked interest the discussion upon anæsthetics in the St. Louis society, and although there is some discrepancy of opinion yet it is of more than usual interest. I have used anæsthetics in parturition to some extent, but always with due caution; and to state the facts, I prefer, in natural labor, to submit the case without medication of any kind. In April, I waited on Mrs. B——, in her sixth confinement. It was a breech presentation. The os being rigid, and being appealed to by her, I administered chloroform during each pain, but not in sufficient amount to complete stupor. The breech and trunk were born in the course of a few hours, and just the moment the neck reached the external genitalia the pains ceased completely, and with all known methods to establish them again not a pain came till about twenty minutes had elapsed. The consequence was complete asphyxia of the child, with no chance of resuscitation.

Now, I attribute the cessation of these pains to the use of chloroform. I may be wrong; if I am, I will submit to being criticised, but this is the conclusion I have arrived at. Here was the death of the child, and had I been a "*new beginner*" I would have been censured, and the death of the child would bave been attributed to my "*negligence*" or "*ignorance*," or both. Hence, I advise young physicians to use anæsthetics only in extreme cases—threatened convulsions or manual delivery—and let natural labors rest with nature's aid. As to ether, I deem it as unsafe as chloroform. I am satisfied, after considerable experience with it, that there is more danger of paralysis of the lungs from its use than from chloroform, and I *know* that it is better calculated to embarrass the physician than chloroform, for its effects are not so quick nor so complete.

I give this for what it is worth. They are simply ideas gathered from close observation during active duty in the practice of medicine.—*Obstetric Gazette.*

